# Low risk of relapse and deformity among leprosy patients who completed multi-drug therapy regimen from 2005 to 2010: A cohort study from four districts in South India

**DOI:** 10.1371/journal.pntd.0009950

**Published:** 2021-11-23

**Authors:** Prabu Rajkumar, Girish Kumar Chethrapilly Purushothaman, Manickam Ponnaiah, Devika Shanmugasundaram, Jayasree Padma, Rang Lal Meena, Selvaraj Vadivoo, Sanjay M. Mehendale

**Affiliations:** 1 Division of Health Systems Research, ICMR-National Institute of Epidemiology, Chennai, India; 2 Laboratory Division, ICMR-National Institute of Epidemiology, Chennai, India; 3 Division of Online Courses, ICMR-National Institute of Epidemiology, Chennai, India; 4 Division of Epidemiology and Biostatistics, ICMR-National Institute of Epidemiology, Chennai, India; Adolfo Lutz Institute of Sao Jose do Rio Preto, BRAZIL

## Abstract

**Introduction:**

Relapse of leprosy among patients released from treatment (RFT) is an indicator of the success of anti-leprosy treatment. Due to inadequate follow-up, relapse in leprosy patients after RFT is not systematically documented in India. Relapsed leprosy patients pose a risk in the transmission of leprosy bacilli. We determined the incidence of relapse and deformity among the patients RFT from the leprosy control programme in four districts in South India.

**Methods:**

We conducted two follow-up surveys in 2012 and 2014 among the leprosy patients RFT between 2005 and 2010. We assessed them for any symptoms or signs of relapse, persistence and deformity. We collected slit skin samples (SSS) for smear examination. We calculated overall incidence of relapse and deformity per 1000 person-years (PY) with 95% confidence intervals (CI) and cumulative risk of relapse.

**Results:**

Overall, we identified 69 relapse events, 58 and 11, during the first and second follow-up surveys, respectively. The incidence of relapse was 5.42 per 1000 PY, which declined over the years after RFT. The cumulative risk of relapse was 2.24%. The rate of deformity among the relapsed patients was 30.9%. The overall incidence of deformity was 1.65 per 1000 person years. The duration of *M*. *leprae* detection in smears ranged between 2.38 and 7.67 years.

**Conclusions:**

Low relapse and deformity rates in leprosy RFT patients are indicative of treatment effectiveness. However, a higher proportion of detection of deformity among relapsed cases is a cause for concern. Periodic follow-up of RFT patients for up to 3 years to detect relapses early and ensure appropriate treatment will minimize the development of deformity among relapsed patients.

## Author summary

India achieved leprosy elimination (<1 case/10000 population) in 2005. However, it remains a country with a high burden of leprosy (120,334 cases in 2017–18), with an annual detection of more than 10000 new cases, including children, indicating widespread active transmission. In addition to the untreated leprosy cases, even patients who relapse after release from leprosy treatment play a significant role in disease transmission. Early detection and management of leprosy relapse cases will minimize the disease transmission and might prevent leprosy-associated deformity. Leprosy management services are primarily provided by the government healthcare system in India and supported by several major non-governmental charitable organizations. The burden of leprosy relapse in India cannot be measured since there is no provision to follow-up the patients who complete multi-drug treatment (MDT) in the national leprosy program. We report the incidence of leprosy relapse among the patients who completed leprosy treatment from the national leprosy control program in four districts in South India. The report also highlights the effectiveness of MDT in program settings, the lower risk of developing deformity after treatment completion, and the need to incorporate an appropriately designed follow-up strategy.

## Introduction

The primary focus of leprosy control programmes worldwide is to detect and treat leprosy cases, stop disease transmission and prevent the development or worsening of disabilities [1]. The National Leprosy Eradication Programme (NLEP) of India uses WHO recommended multi-drug therapy (MDT) for leprosy patients as part of general health services. On completion of the fixed course of MDT, patients are labelled as released from treatment (RFT) irrespective of their clinical status [2].

The rate of occurrence of relapse among leprosy patients is an important indicator of the success of MDT [3]. Appropriately planned periodic follow-up of the RFT patients is essential for accurate and early identification of relapse in the programmatic settings [4]. Relapse of leprosy after RFT can pose a significant risk in transmission of disease. Hence, early diagnosis of relapses and initiation of appropriate treatment are believed to be critical interventions to minimize further transmission from relapsed cases and prevent complications [5].

The rates of relapse reflect the quality of anti-leprosy services. Research on relapse is considered a priority area by WHO [3,6]. In India, leprosy treatment services are primarily provided by the government health systems and supported by several major non-governmental charitable organizations. The current programmatic limitations in tracking relapses are lack of adequate funding, trained workforce, and motivation for doing appropriate follow-up of the RFT patients [7,8].

The trend of relapse over the years after completion of MDT is a crucial epidemiological indicator, which can help design an appropriate follow-up strategy. The literature on reliable estimates of leprosy relapse under programme conditions is scanty. The available follow-up reports [9–11] are primarily from controlled settings; hence figures will have limited applicability for the national-level leprosy control program mainly because they have been done in an older timeframe.

Reactivation of persistent leprosy bacilli or reinfection or documented secondary drug resistance in RFT patients could play a role in the occurrence of relapse [2,12,13]. The NLEP does not have a mechanism of follow-up; therefore, the risk of development of relapse among the leprosy patients after completion of treatment is not measurable.

An important goal of MDT is to reduce deformity due to leprosy. Many leprosy patients do seek medical care only after the occurrence of deformity. Further, the deformity can occur even after the completion of MDT treatment. Hence, it is essential to formulate appropriate prevention and control strategies for timely detection and treatment of deformities after completion of MDT [14].

ICMR-National Institute of Epidemiology (ICMR-NIE) conducted a prospective cohort study of leprosy patients who completed treatment and were declared RFT during 2005–2010 from four districts in Tamil Nadu and Andhra Pradesh states in South India. The objective of the study was to document the rate of relapse among these RFT patients. Our follow-up in 2012 had provided relapse rates from 1.88 to 7.3 years after RFT. The study methods and findings of the first follow-up have been previously published [5]. Considering the need to explore this cohort of the RFT patients further to identify new occurrences of relapses and deformity, we did another round of follow-up survey during 2013–14. This manuscript provides estimates of the overall incidence of leprosy relapse and deformity among the cohort of RFT patients examined in both the follow-up surveys. Further, we describe the risk factors for relapse, present findings on the bacterial index of slit skin smear (SSS) samples and the status of relapse patients identified during the first follow-up.

## Methods

### Ethics statement

This study was approved by the Institutional Human Ethics Committee (protocol ID # NIE/IEC/2011-006) of ICMR-NIE. Approvals for conducting the study were obtained from the State Leprosy Officers of Tamil Nadu and Andhra Pradesh. Patients were assessed after obtaining their written informed consents. Patients with relapse, persistence and deformity were referred to the nearest public health facility for further evaluation and management.

### Study design, setting and population

We did follow-up surveys among the cohort of leprosy patients RFT between 2005 and 2010 from three districts in Tamil Nadu and one district in Andhra Pradesh states in South India. The demographic, geographic and leprosy indicators were different in these study districts. All the study districts achieved the leprosy elimination; however, leprosy new case detection and deformity (per 100,000 population) were reported maximally in Cuddalore (8.18) and Salem (3.35) districts, respectively [5,15–17]. The RFT patients identified in the first follow-up in 2012 [5] formed the cohort for the second follow-up survey conducted between September 2013 and July 2014.

### Data collection

Fieldworkers with appropriate training and several years of experience in leprosy work conducted the follow-up of the RFT patients by visiting their households. The fieldworkers examined and recorded the status of each case, occurrence and duration of new clinical signs and symptoms of leprosy, including skin, nerve lesions and grade of deformity on a body chart during both the follow-up visits.

The detailed operational definitions were described previously [5]. Study participants who were suspected of having clinical symptoms suggestive of leprosy relapse, reaction or persistent lesions and those requiring further evaluation were referred to the nearest public health care facility for clinical confirmation by the specialist doctors. For bacterial index (BI) assessment, SSS samples were collected from the most prominent lesion or two ear lobes in case of subjects with no visible skin lesions. Participants with a confirmed diagnosis of relapse received MDT as per the WHO recommended guidelines from their local public health service providers.

### Statistical analysis

We analyzed the baseline characteristics, including socio-demographic features, clinical characteristics and leprosy diagnostic classification of the study participants. We calculated person-years (PY) of follow-up individually from the date of RFT until the date of examination and sample collection during the second follow-up in 2013–14 in the present study. We estimated relapse rates per 1000 PY and their 95% confidence intervals (CI). In the absence of appropriate follow-up information such as steroid treatment, we calculated relapse rates by both considering and not considering patients with leprosy reaction as relapse. We compared the relapse rates by gender and types of leprosy using the mid-p exact test. We calculated the cumulative risk of relapse for five years from the estimated rate using the density-based method.

Further, we performed recurrent event multiple regression analysis using the Andersen-Gill method (with the district as stratification variable) to estimate Hazard Ratio and their 95% CI for factors associated with relapse after RFT. We used the computed relapse rates to estimate the expected number of relapses in patients who could not be examined. We calculated the deformity rate per 1000 PY and 95% CI for the relapsed patients with deformity.

We evaluated the current status of nerve lesions, deformity, and relapse observed during first follow-up in the present follow-up. We also described the current status of persistent leprosy patients identified during the first follow-up survey. We used STATA V 14.0 (Stata Corp, College Station, Tx, USA; 2015) for data analysis.

### Laboratory analysis

The skin smear was stained using the Ziehl-Neelsen method, and the bacteriological index (BI) was recorded as per Ridley’s logarithmic scale [18,19].

## Results

### Profile and follow-up status of the cohort

**Fig 1:**
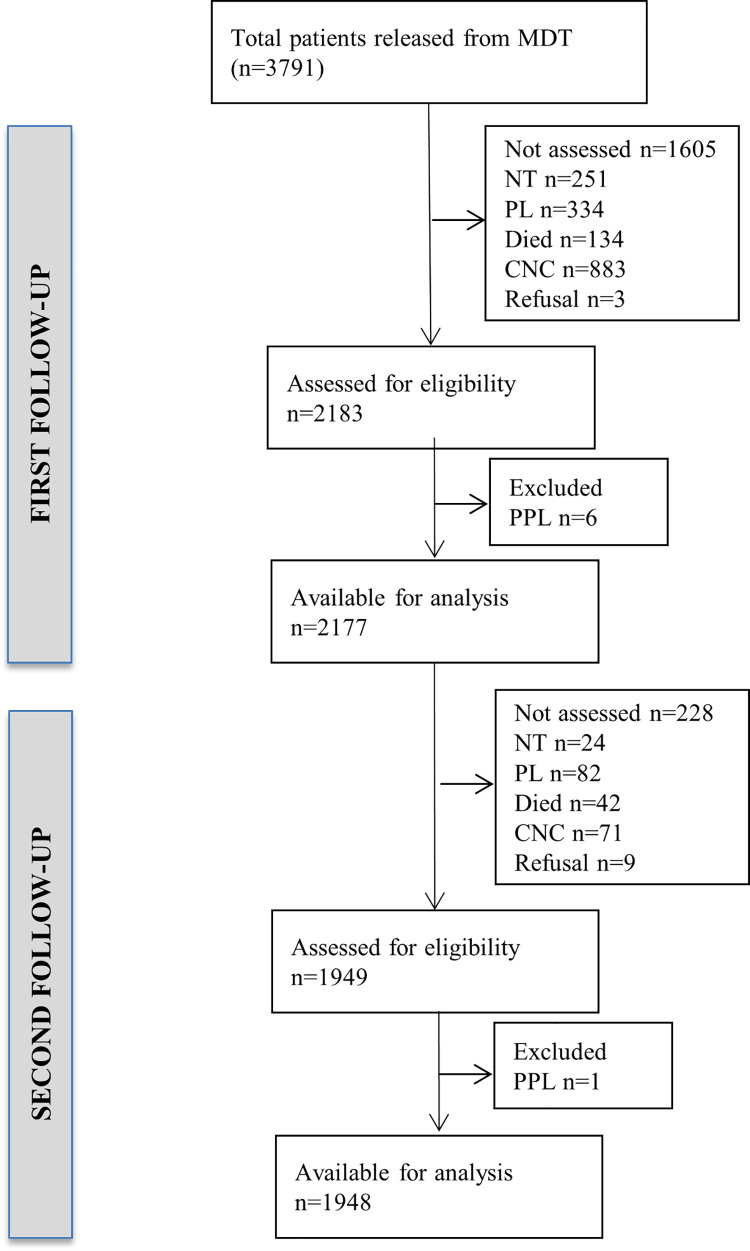
Recruitment process of RFT patients in first and second follow-up surveys in study districts: Relapse and deformity among patients released from National Leprosy Eradication Programme of India: A cohort study from four districts in South India. RFT–Released from treatment; NT–Not traceable; PL–Permanently left; CNC–Could not be contacted; PPL–Patients with persistent leprosy.

Out of 3791 patients declared RFT from the general health services between April 2005 and March 2010, the fieldworkers examined 2183 (58%) in the first follow-up survey in 2012. Due to various reasons, 1608 (42%) RFT patients could not be assessed during the first survey. Of the 2183 RFT patients covered in the first follow-up, 1210 (55%) were males and 1277 (59%) from PB group. Of the participants examined, 775 (36%) were illiterate, and 1404 (64%) were married. During the first follow-up visit, six RFT patients had lesions suggestive of persistent leprosy and were excluded from the calculation of relapse. Of the 2177 leprosy patients eligible for evaluation in the second follow-up survey, 228 (11%) RFT patients could not be assessed, and field workers examined 1949 (90%) participants. **(Fig 1)** One patient with persistent lesion identified during the second follow-up was excluded from further analysis. Finally, 1948 patients were available for analysis during the second follow-up survey. The cumulative median follow-up period was 72 months [Range: 42–111 months].

### Incidence of relapse

Overall, we identified 68 patients to have relapsed (corresponding to 69 total relapse events; 58 at first and 11 at second follow-up). The number of relapse events was 31 and 38 in MB and PB RFT patients, respectively. **(Table 1)** We classified the cases of reaction as relapse due to non-availability of required follow-up data. Thus, the overall incidence of relapse was 5.42 per 1000 PY (95% CI = 4.28 to 6.87). The relapse rate (per 1000 PY) was higher among males (6.72) than females (3.84) [p = 0.0277]. Relapse rate was slightly higher among MB than among PB (5.95 vs 5.06; p = 0.5002) leprosy patients. **(Table 2).**

**Table 1:** Distribution of RFT patients and relapse events: Relapse and deformity among patients released from National Leprosy Eradication Programme of India: A cohort study from four districts in South India.

	MB		PB		Total	
RFT year	N	Relapse	N	Relapse	N	Relapse
2005	63	0	127	2	190	2
2006	184	2	261	7	445	9
2007	159	4	246	3	405	7
2008	201	5	268	8	469	13
2009	238	16	301	17	539	33
2010	57	4	72	1	129	5
Total	902	31	1275	38	2177	69

**Table 2:** Number and incidence of relapse (per 1000 person-years) among the RFT patients: Relapse and deformity among patients released from National Leprosy Eradication Programme of India: A cohort study from four districts in South India.

Characteristics		Relapse (n)	RFT patients (N)	Relapse Rate per 1000 person-years
**Overall**		**69**	**2177**	**5.42**
Age at first follow-up (years)	≤ 14	3	150	3.69
	15–35	28	781	6.13
	36–50	23	614	6.33
	> 50	15	632	4.04
Gender	Male	47	1206	6.72
	Female	22	971	3.84
Type of leprosy	MB	31	902	5.95
	PB	38	1275	5.06
Year of release from treatment	2005	2	190	1.3
	2006	9	445	2.75
	2007	7	405	2.73
	2008	13	469	5.15
	2009	33	539	13.99
	2010	5	129	10.47

**Fig 2:**
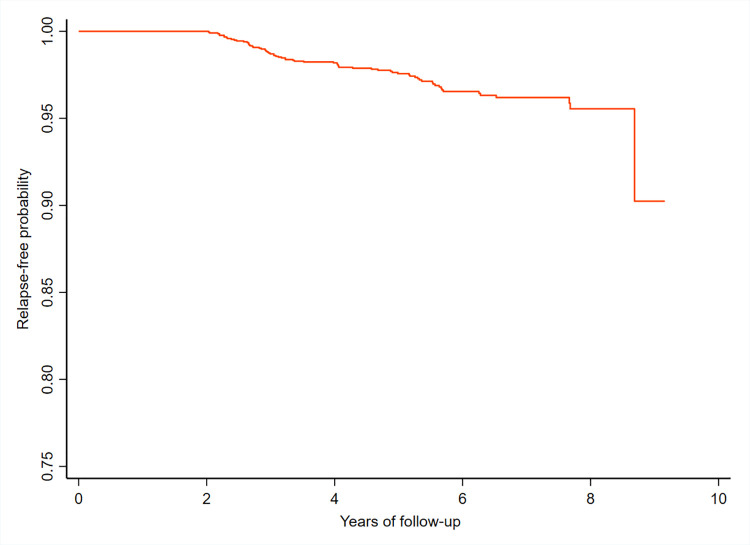
Survival curve by Kaplan Meier method–Relapse free probability among RFT patients: Relapse and deformity among patients released from National Leprosy Eradication Programme of India: A cohort study from four districts in South India.

The incidence of relapse was higher immediately after release from the treatment. The five-year cumulative risk of relapse was 2.24%. The regression analysis indicated that risk was higher for male gender [Hazard Ratio: 1.68; 95% CI = 1.01 to 2.79)], whereas age and type of leprosy were not significantly associated with the relapse. **(Table 2)** We did not observe any significant difference in the relapse rates between the study districts. The relapse rate by not considering reaction cases as relapse (five in the first and one in the second follow-up rounds, respectively) was 4.95 per 1000 person-years (95% CI = 3.87–6.34). The survival curve (based on Kaplan Meier method) shows the cumulative probability of people free from relapse according to the follow-up period **(Fig 2)**. We found that the probability of relapse after RFT in the first five years of follow-up was only 2.4%.

### Profile of relapse patients

Among the relapses that occurred during both rounds of follow-up survey, recurrent lesions [n = 42], new lesions [n = 21] and reactions [n = 6] were observed. Nerve lesions ranging between 1 and 10 were observed in 39 relapsed patients (MB = 23, PB = 16). Overall, 51 (74%) relapses occurred within three years, and 67 (97%) occurred within five years of RFT.

### Deformity status of the relapsed patients

Out of 68 patients with relapse, 21 (31%) had a deformity, of which 13 (62%) had grade 2 deformity. **(Table 3)** The overall deformity rate was 30.9%. The incidence rates of deformity were 3.07 and 0.67 per 1000 PY among the MB and PB patients, respectively. The overall incidence of deformity was 1.65 per 1000 PY (95% CI = 1.08 to 2.53).

**Table 3:** Deformity among the RFT patients with relapse: Relapse and deformity among patients released from National Leprosy Eradication Programme of India: A cohort study from four districts in South India.

First follow-up	Second follow-up			
	Grade 0	Grade 1	Grade 2	Total
Grade 0	47	2	1	50
Grade 1	6	0	1	7
Grade 2	10	0	1	11
Total	63	2	3	68

### Bacterial index

Of the 68 relapse patients, we collected smear samples from 64 (94%) and six (9.4%) of them were smear positive. All the smear positives were from MB (BI range = 0.25 to 4.5). The duration of positivity ranged between 2.38 years and 7.67 years after RFT.

### Status of patients with relapse diagnosed in the first follow-up

We were able to examine 52 (90%) RFT patients who were diagnosed with leprosy relapse in the first follow-up. Among them, one patient had lesions suggestive of persistent leprosy during the second follow-up evaluation, whereas another patient had developed repeated skin lesions after completing the entire course of PB MDT during the period between the first and second follow-up visits. The latter was diagnosed as repeat relapse in the very same patient. Out of 18 patients who had a deformity in the first follow-up, we observed only two patients with deformity in the second follow-up. One patient had grade 1 in the first follow-up developed grade 2 in the second follow-up. The second patient remained in grade 2 in both the follow-up visits.

### Sensitivity analysis

The loss to follow-up in the study cohort was between 6.1% and 23.7%. Based on the assumption of missing completely at random, we estimated that expected relapses among these lost-to-follow-up patients could be from 43 to 92 [N = 1839; first round = 1608; second round = 231].

### Profile of patients with persistent leprosy lesions

Overall seven RFT patients were identified with persistent lesions during the follow-up visits (six and one in the first and second follow-up visits, respectively). Of the patients with persistent leprosy, four were from the MB group, and four were males. Among persistent leprosy patients, two each had grade 1 and 2 deformities. Six cases that underwent SSS examination were negative. We examined 3 (50%) available persistent cases from the previous follow-up, and none had any signs of relapse or deformity.

## Discussion

We did two rounds of surveys among a cohort of leprosy patients released from India’s National Leprosy Eradication Programme in four districts in South India and documented a low risk of relapse over six years post-RFT. This observation has direct relevance to NLEP because it very clearly signifies the importance of using WHO-recommended MDT as an effective method for leprosy treatment.

A study from India reported that approximately 24% of the relapses diagnosed in the first 18 months after RFT could be late reversal reactions [20]. The relapse rates were very low even after considering the reaction cases as relapses, which further decreased in the second follow-up round in our study. The relapse rate reported in studies from India and other countries ranged from 0.73 to 19.7 per 1000 PY [9–11,14,21–25]. Wide variation in the relapse rates has been previously reported. This could be due to differences in the definition of relapse, skin smear positivity range, treatment duration, study settings, and follow-up strategy [2,5,26]. Our estimates could be a suitable comparator for settings wherein leprosy programmes have used similar definitions.

We observed a declining trend in the relapse rates over the years after RFT, which became negligible after five years. This finding is in line with the trend of relapse in the first follow-up [5]. A retrospective analysis among patients RFT from South India reported a similar trend, with most patients relapsing within three years [9]. The findings from the present community-based study will be helpful to NLEP in designing the strategies for follow-up of RFT patients to enable timely capture of relapse events. Appropriate documentation of late relapse after RFT is also an important indicator to assess the effectiveness of leprosy MDT. Late relapse can occur even up to 16 years after RFT, as documented in a study from the Philippines, which underscores the need for a longer follow-up.[27]

We observed that men had a significantly higher rate of relapse and this finding was consistent with our earlier observation [5]. A study in North India reported no significant gender-wise difference in relapse rates [11]. Male gender, inadequate/irregular therapy, high initial bacterial index, and the number of skin and nerve lesions are reportedly associated with a higher rate of relapse [2]. A study from China reported a higher rate of relapse among PB RFT patients [22]. The five-year cumulative risk of relapse observed in our study was higher than previously reported [4,9]. The probability of relapse in the first five years of follow-up observed in our study was much lower than a retrospective cohort study conducted in Columbia. The higher probability of relapse reported in that study could be due to retrospective study design, target patients (MB), higher exclusion (70.4%) and relapse (67 per 1000 PY) rates [28].

Skin smear positivity after seven years of RFT documented in our study indicates the potential of continued transmission of *M*. *leprae* to contacts. This finding emphasizes the need for periodic evaluation of RFT patients to detect recurrence of leprosy symptoms. Other studies have also documented skin smear positivity after RFT [9,29]. The positivity might be due to the reactivation of dormant bacteria or reinfection with *M*. *leprae* [11,30].

With appropriate referral to the public health facility and treatment, most of the relapse patients identified during the first follow-up did not show any signs of leprosy in the second follow-up. This finding highlights the importance of building a follow-up strategy for early detection and treatment of relapses. The occurrence of repeat relapse in a PB RFT patient, though rare, is a matter of concern [31].

Data on leprosy deformity are reported as proportions, and incidence rates are not usually reported [14,32,33]. The overall incidence of deformity observed in our study is lower than that reported from Malawi (5 per 1000 PY) [32]. The deformity rate among MB RFT patients is also lower than the rates reported from North India (2.69 per 100 PY) and Brazil (4.5 per 100 PY) [14,34]. The possible reason for such a difference could be that the studies conducted among the RFT patients were from defined cohort or clinic-based settings where more rigorous follow-ups were undertaken. The status of the patients identified with deformity in the first follow-up improved after appropriate management. However, the proportion of deformity identified during the second follow-up remained similar to the first follow-up, which is a matter of concern. Late diagnosis and delay in initiation of appropriate management strategies were reported as major risk factors for development disability [33]. Therefore, early diagnosis of deformity and providing adequate awareness are necessary to prevent permanent damage.

### Limitations

Our study has a few limitations. We could not document the exact time of occurrence of symptoms/signs of relapse and deformity and actual duration of *M*. *leprae* positivity due to the limitations of the study design and absence of slit-skin smear findings at RFT in the programme. A higher proportion of lost-to-follow-up might affect the validity of the relapse estimates presented in our study. However, their baseline characteristics were comparable with those examined and based on sensitivity analysis, the observed number of relapses was within the expected range. There is a chance that our observation is an underestimate since the highest value of the expected number of relapses was 92.

### Conclusion

We documented a very low relapse rate, which decreased over years among the patients released from the leprosy control programme. The low deformity rate among the RFT patients is also an important indicator of the effectiveness of NLEP. However, the proportion of deformity among the relapsed patients and persisting smear positivity need special attention.

### Recommendations

The leprosy control program of India needs to design a follow-up mechanism considering the limited availability of a trained workforce for early diagnosis and management of relapse among RFT patients. Rigorous case identification and ensuring treatment completion are essential to minimize the occurrence of relapse among the RFT patients. Further, appropriate counselling needs to be provided to the RFT patients for early reporting of relapse at the nearest health facility for initiating treatment to minimize permanent damage. A follow-up strategy using digital technologies may be helpful in resource-limited settings.
